# Exploring the basis of Traditional Chinese Medicine in treating cardiovascular disease: insights into the microbiota-gut-heart axis

**DOI:** 10.1186/s13020-026-01336-w

**Published:** 2026-02-03

**Authors:** Xinxin Hou, Xiaoqi Guan, Xiaoyun Qin, Hai-dong Guo

**Affiliations:** Academy of Integrative Medicine, Shanghai University of Traditional Chinese Medicine, Shanghai, 201203 China

**Keywords:** Gut microbiota, Traditional Chinese Medicine, Cardiovascular disease, Gut–heart axis, Pharmacomicrobiology

## Abstract

Despite being widely used to treat cardiovascular disease (CVD), there are still limitations of Traditional Chinese Medicine (TCM) for their widespread use due to undetermined pharmacological mechanisms. The emerging area of “pharmacomicrobiology” facilitates the pursuing of the complex interactions between TCM and gut microbiome as the gut microbiota influences TCM bioavailability and efficacy, vice versa TCM affects the microbial composition and function. Furthermore, research over the past decades has proved that gut dysbiosis and diverse microbial metabolites intimately related to the onset and progression of CVD. Herein, we summarize latest understanding of the TCM–gut–heart axis in CVD, with an emphasis on describing microbial changes and the associated mechanisms in CVD, elaborating the role of gut microbiota in metabolizing TCM, and how TCM can be used to treat CVD through affecting the gut microbiota. We further highlight the challenges and future directions that target TCM–gut–heart axis in prospects for personalized medicine in CVD, thus to present novel insights for developing new therapeutic approaches.

**Graphic Abstract:**

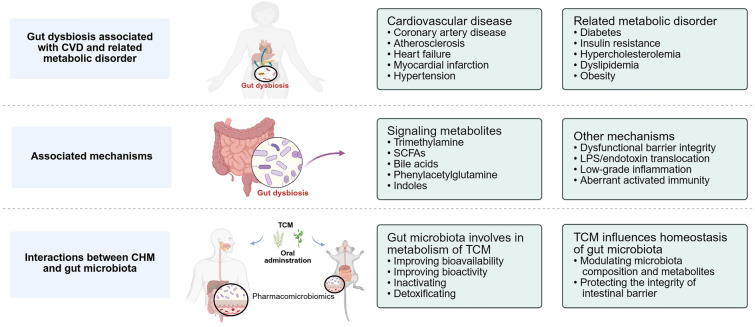

## Introduction

Cardiovascular disease (CVD) persists as the predominant cause of global mortality and disability, despite decades of abundant efforts and significant advancements towards early detection and targeted therapies [1, 2]. The escalating burden of CVD is exacerbated by synergistic risk factors (hypertension, dyslipidemia, obesity, diabetes) and the limitations of conventional Western therapies (interindividual heterogeneity, adverse effects) [3]. Thus, novel therapeutic targets and approaches for a better prevention and treatment of CVD are warranted.

Recently, researchers have begun to decipher the complex pathophysiology of CVD, which involves multifactorial processes such as inflammation and immune-mediated pathways [4], autophagic or mitophagic defects [5], and endothelial dysfunction [6]. Owing to the rapid development of novel omics approaches such as metagenomics, metatranscriptomics and metaproteomics, emerging pathogenic paradigms, including the gut–heart axis, highlight the gut microbiota (known as the “second genome”) as a key mediator of CVD pathogenesis [7–13]. For example, in the Framingham Heart Study, cholesterol-metabolizing bacteria *Oscillibacter* species that encode for conserved cholesterol-metabolizing enzymes, was found to be associated with CVD [14]. More recently, by analyzing metagenomic datasets from a high-fiber dietary intervention in type 2 diabetes and 26 case–control studies across 15 diseases, including CVD, researchers have found a core microbiome structure of health-relevant gut microbes, offering novel targets for health biomarkers and disease modulation [15]. Taken together, these evidences link gut microbial perturbations to CVD pathogenesis and progression, which may serve as a promising precise intervention target for CVD.

Traditional Chinese Medicine (TCM), with the advantages of multiple targets, pathways, and components, has shown observable effects in the treatment of CVD and exhibits high safety and no serious adverse effects [16–18]. Recent high-quality randomized controlled clinical trials (RCTs) have provided evidence for the application of TCM in CVD: trials on the Qili Qiangxin capsule for use in combination therapy of chronic heart failure showed that Qili Qiangxin reduced cardiovascular death and/or first hospitalization for heart failure compared to a placebo (25.02% versus 30.03%) [19]; trails on Tongxinluo for the adjunctive treatment of acute ST-segment elevation myocardial infarction showed that Tongxinluo reduced 30-day major adverse cardiac and cerebrovascular events (MACCEs) compared to placebo (3.4% versus 5.2%) [20]; and trails on the Songling Xuemaikang capsule for the treatment of grade 1 hypertension showed that it demonstrates superiority in reducing 24-h systolic/diastolic blood pressure compared to a placebo (− 5.91 ± 11.32/− 4.66 ± 9.33 mm Hg versus − 2.26 ± 10.47/− 2.56 ± 8.76 mm Hg) [21]. However, the pharmacological mechanisms of TCM remain to be fully elucidated. Notably, gut microbiota investigations have emerged as a cutting-edge approach for unraveling the scientific basis of TCM, linking the mechanisms of TCM to CVD treatment [22]. On the one hand, gut microbiota mediates the metabolism or biotransformation of the active ingredients of TCM, and the resulting metabolites enhance pharmacological activity and/or improve bioavailability. On the other hand, TCM can alter the composition and structure of the gut microbiota as well as bacterial metabolite levels, thus exerting regulatory effects on the intestinal barrier, immune system, and systemic metabolism.

In this review, we summarize the microbial changes in CVD-relevant phenotypes and their underlying mechanisms in promoting CVD, focusing on microbiota-derived metabolites. We then discuss herb–microbiota interactions, elaborating the role of the gut microbiota in metabolizing TCM and the complex mechanisms of TCM efficacy in the regulation of gut microbes in CVD therapy. Finally, we highlight therapeutic interventions targeting the gut microbiome, along with TCM usage, as a potential treatment for CVD. This tripartite framework links mechanistic insights to clinical uses and unveils a new path for cardiovascular treatments.

## Gut microbiota and host cardiovascular health

A healthy gut microbiota is dominated by anerobic bacteria of the phyla Firmicutes and Bacteroidetes, which support host homeostasis via metabolic, immunomodulatory, and epithelial maturation functions [23–26]. The gut–heart axis, an emerging research area regarding the intricate interplay between cardiovascular and gastrointestinal systems, has attracted immense attention recently. The gut microbiome is considered a key metabolic organ that communicates with distal organs through its derived metabolites, which serve as mediators of microbial influence on the host [27]. Common metabolites such as short-chain fatty acids (SCFAs) have been shown to facilitate host blood pressure homeostasis, ameliorate myocardial damage, and improve glucose and lipid metabolism, thus lowering the risk of CVD [28–30]. As the mammalian intestinal environment varies according to host hygiene, diet, and drug use, the microbiome can rapidly change due to alterations in either the composition of the microbial community or individual microbial genomes, with various consequences for host physiology [31]. Therefore, modulation of gut microbiota may benefit cardiovascular function and studying these modulations may lead to the discovery of new therapeutic approaches and strategies for improving cardiovascular health.

## Microbial dysbiosis in cardiovascular pathogenesis

There is growing evidence that gut dysbiosis contributes to adverse phenotypes such as aberrant metabolic conditions and the development of CVD [32]. In large metagenome-based cross-sectional studies to assess the relationships between the gut microbiome, plasma metabolome and CVD risk, bacterial pathways of l-methionine biosynthesis were identified to be associated with atherosclerosis and metabolic risk scores of CVD [33]. In addition, the gut microbiota is capable of identifying the risk of new MACCEs, thus helping with secondary prevention of cardiovascular events. A higher abundance of the Firmicutes phylum, Lactobacillaceae family, and *Lactobacillus* genus and a lower abundance of the Bacteroidota phylum, Bacteroidia class, and Bacteroidales order were observed in patients with MACCEs compared with non-MACCE patients [34]. We summarize the recent advances in the understanding of the microbial signatures in CVD and related cardiometabolic phenotypes in Table 1.

**Table 1:** The gut microbiota profile in CVD and related cardiometabolic phenotypes

Diseases/phenotypes	Gut microbiota changes	Associated mechanisms	Study design	References
Coronary artery disease	Increased: *Bacteroides*, *Clostridium cluster* XI, *Clostridium subcluster* XIVa Decreased: *Lactobacillales*	NA	Case–control study involving 39 patients and 30 controls with coronary risk factors	[35]
	Increased: *Firmicutes phylum* Decreased: *Bacteroidetes phylum*	Through gut microbial metabolite TMAO	Case–control study involving 29 patients and 35 healthy controls	[36]
	Increased: *Escherichia-Shigella*, *Enterococcus* Decreased: *Faecalibacterium*, *Roseburia, Subdoligranulum*, *Eubacterium rectale*	Enhanced amino acid metabolism, phosphotransferase system, propanoate metabolism, LPS biosynthesis, and protein and tryptophan metabolism	Case–control study involving 70 patients and 98 healthy controls	[37]
Atherosclerosis	Increased: *Escherichia*, *Streptococcus* Decreased: *Bacteroide*,* Prevotella*	Increased pro-atherosclerotic metabolite TMAO; decreased SCFAs, particularly butyrate	Case–control study involving 218 patients and 187 healthy controls	[11]
	Increased: *Collinsella* Decreased: *Roseburia*, *Eubacterium*	Peptidoglycan synthesis genes enriched in patients; phytoene dehydrogenase genes enriched in controls	Case–control study involving 12 patients with symptomatic atherosclerotic plaques and 13 controls without large vulnerable plaques in the carotid arteries	[38]
Heart failure	Decreased: *Coriobacteriaceae*, *Erysipelotrichaceae*, *Ruminococcaceae* and *Blautia*	NA	Case–control study involving 20 patients and 20 healthy controls	[39]
	Decreased: *Eubacterium rectale*,* Dorea longicatena*	Through gut microbe-derived metabolites such as SCFAs	Case–control study involving 12 patients and 12 healthy controls	[40]
	Increased: *Prevotella*, *Hungatella*,* Succinclasticum* Decreased: *Lachnospiracea* family (including *Anaerostipes*, *Blautia*, *Coprococcus*, *Fusicatenibacter*, *Lachnospiraceae* FCS020/ NC2004/ ND3007, *Pseudobutyrivibrio*, *Eubacterium hallii* group), *Faecalibacterium*,* Bifidobacterium*	Depletion butyrate-producing bacteria; inverse correlation with Treg	2 independent cross-sectional cohorts of patients (discovery, n = 40; and validation, n = 44) and healthy controls (n = 266, randomly allocated to the 2 cohorts for comparison)	[41, 42]
	Increased: *Ruminococcus gnavus* Decreased: *Faecalibacterium prausnitzii*	Stimulated bacterial antigen-specific Th1 and Th17 immune responses; aggravated chronic inflammation	Case–control study involving 53 patients and 41 controls	[43, 44]
Hypertension	Increased: *Prevotella*, *Klebsiella*, *Enterobacter*,* Fusobacterium*	Through altered microbial richness, diversity, and function associated with inflammation and immune response	Cohort study of 41 healthy controls, 56 pre-hypertension individuals, 99 hypertension patients	[45]
	Decreased: *Eubacterium rectale*, *Rosebria* and other major butyrate-producing genera	Impaired gut epithelial barrier function; increased intestinal inflammation and permeability (increased plasma levels of zonulin, I-FABP, LPS, and Th17 cells)	Cohort study of 60 healthy controls, 60 patients; Mechanism validated in chronic angiotensin II infusion mouse model	[46]
	Increased: *Klebsiella spp.*, *Streptococcus spp.*, *Parabacteroides merdae* Decreased: *Roseburia spp.*,* Faecalibacterium prausnitzii*	Higher membrane transport, LPS biosynthesis, and steroid degradation metabolic signaling signature of patients; higher metabolism of amino acids, cofactors, and vitamins in control	Case–control study involving 60 patients and 60 healthy controls	[47]
	Increased: *Anaerovorax*, *Clostridium* IV*, Oscillibacter*, *Sporobacter*, *Catabacter*, *Robinsoniella*, *Parasporobacterium*	Systemic inflammation; production of TMAO	Cross-section study in 529 middle-aged adults from CARDIA (Coronary Artery Risk Development in Young Adults) study	[48]
	Increased: *Alistipes finegoldii*,* Lactobacillus spp.* Decreased: *Prevotella spp.*,* Clostridium spp*	Decreased microbial SCFAs and receptor GPR43	Cross-section study involving 23 patients and 46 controls	[49]
	Increased: *Eubacterium rectale* Decreased: *Bacteroides thetaiotaomicron*, *Bifidobacterium*	NA	Case–control study involving 94 patients and 94 controls	[50]
	Negative associations of *Lactobacillus* species with sodium intake and blood pressure	NA	Representative cohort study of 6953 individuals	[51]
Myocardial infarction	Increased: *Streptococcus salivarius*,* Klebsiella pneumoniae* Decreased: *Roseburia hominis*	Increased fecal acetate and butyrate; Decreased serum choline and carnitine	Case–control study involving 117 acute myocardial infarction patients and 78 controls	[52]
	Oral/gut *Streptococcus* and oral *Veillonella* enriched in AMI and correlated with clinical parameters; oral/gut *Neisseria*, *Porphyromonas*, *Bacteroides* and *Ottowia *enriched in healthy control	NA	Case–control study with 37 acute myocardial infarction patients and 36 controls	[53]
CVD and incident events (myocardial infarction, stroke, or death)	Gut microbial metabolite PAGln associated with CVD risk	Induced platelet hyperactivation and thrombosis via α2A, α2B, or β2 adrenergic receptors	Initial discovery cohort study (n = 1,162) and independent validation cohort study (n = 4,000)	[54, 55]
Metabolic dysregulation, obesity and hypertension	Increased: *Faecalibacterium prausnitzii*, *Roseburia faecis*, and other *Clostridiales* Decreased: *Akkermansia muciniphila*, *Alistipes finegoldii*, *Bacteroides*, *Christensenellaceae*, *Methanobrevibacter*,* Oscillospira*	Higher fecal SCFAs; Enhanced gut permeability and inflammation	Cross-section observational study involving 441 individuals	[56]

CVD, cardiovascular disease; TMAO, trimethylamine N-oxide; LPS, lipopolysaccharide; SCFA, short-chain fatty acid; AMI, acute myocardial infarction; Treg, regulatory T cells; PAGln, phenylacetylglutamine

NA: not applicable

**Fig. 1:**
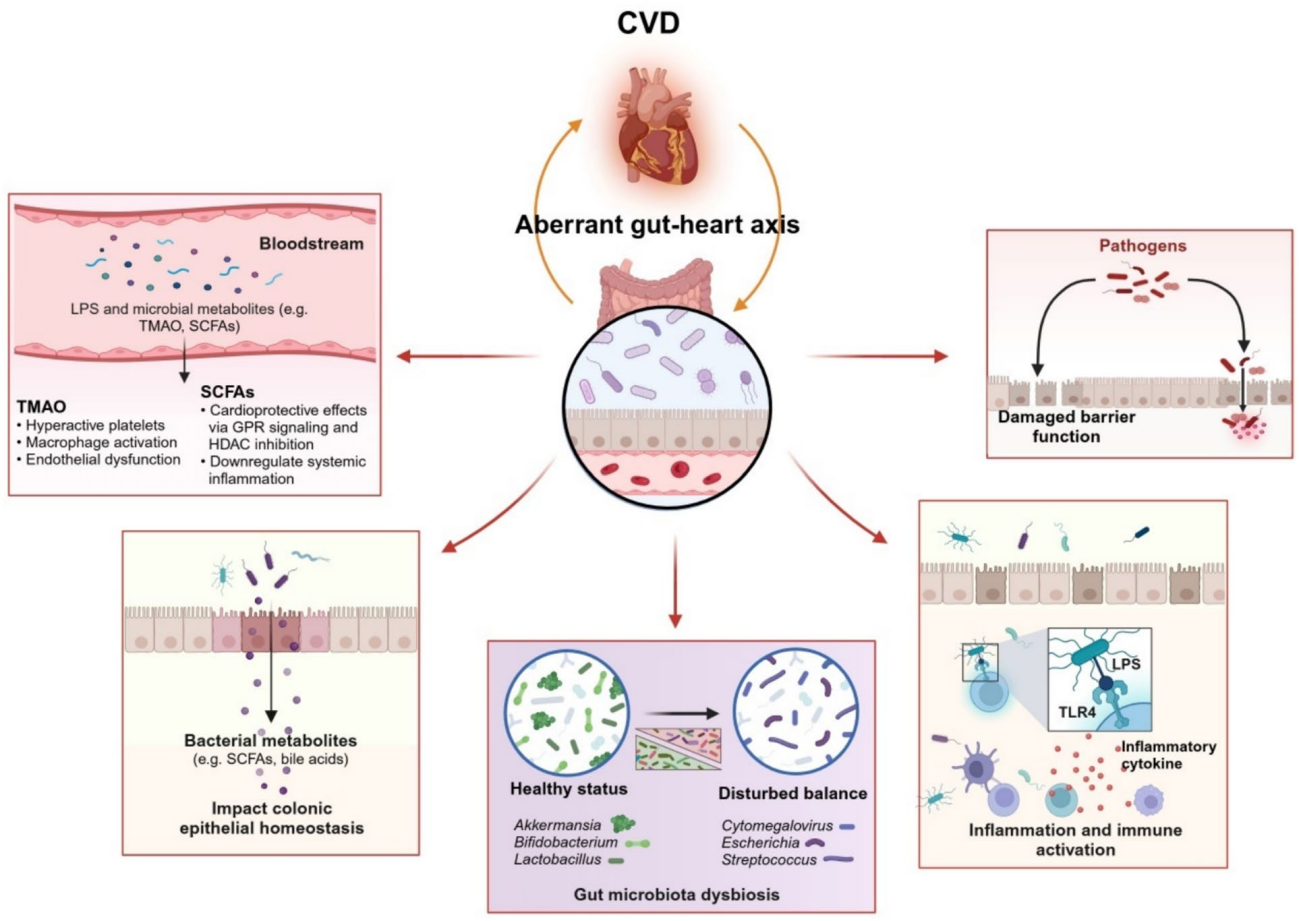
Aberrant gut-heart axis in cardiovascular disease. Cardiovascular disease (CVD) can result in gut dysbiosis while an imbalance of gut microbiota was associated with CVD through multiple mechanisms. Cardiovascular disease (CVD); Lipopolysaccharide (LPS); Toll-like receptor 4 (TLR4); Short-chain fatty acid (SCFA); Trimethylamine-N-oxide (TMAO); G-protein-coupled receptor (GPR); Histone deacetylase (HDAC).

Several mechanisms, such as dysfunctional barrier integrity (namely “leaky gut”), systemic inflammation and endotoxemia, and multiple bacteria-derived pathogenic metabolites [33, 57, 58], have been illustrated in the aberrant “gut–heart” axis (Fig. 1). Dysbiosis results in the translocation of microbes and microbially derived products into circulation [59]; for instance, bacterial lipopolysaccharide (LPS) can translocate into circulation and cause low-grade, non-septic endotoxemia. LPS has been detected in atherosclerotic plaque rather than in atherosclerosis-free arteries, eliciting a pro-inflammatory response via interaction with TLR4, which can promote atherosclerosis progression and thrombus formation [60]. Herein, we focus on presenting the gut microbiota-derived metabolites involved in CVD.

### Trimethylamine, TMAO, and associated metabolites

The gut microbiota can metabolize dietary choline [61], L-carnitine [62], and phosphatidylcholine [63] to produce TMAO, a metabolite closely linked to CVD [64, 65]. Wang et al. first identified TMAO as a potential promoter of atherosclerosis and found that it can not only predict the risk of CVD in a clinical cohort but can also promote atherosclerosis in *Apoe*^−/−^ mice through accelerating macrophage foam cell formation [61]. TMAO promotes vascular inflammation and enhances trained immunity via ER stress/mitochondrial ROS/glycolysis pathways [66]. TMAO also induces myocardial hypertrophy and fibrosis by stimulating the Smad3 signaling pathway [67]. Additionally, TMAO can function as multiple distinct agonists to enhance platelet hyperresponsiveness, thus increasing thrombosis potential [68]. Gut microbial *cutC* (*choline utilization*) has been identified as the major microbial gene responsible for trimethylamine (TMA)’s transformation from choline [69]. Germ-free mice colonized with the engineered commensal *Clostridium sporogenes* with the functional microbial *cutC* gene exhibited elevated circulating TMA and TMAO levels, which directly increase the potential of thrombus formation, proving causality [70]. These findings demonstrate the critical role of the gut microbiota–TMAO axis in CVD pathogenesis, providing novel insights into the interplay among dietary nutrients, gut microbial metabolism, and cardiovascular health. Hence, interventions targeting TMAO accumulation may be a promising strategy to reduce cardiovascular risk.

### Short-chain fatty acids

SCFAs are generally considered beneficial to host cardiovascular health. Clinical and preclinical studies have linked SCFA deficiency and reduced abundance of SCFA-producing bacteria (*Akkermansia*, *Oscillibacte*r, etc.) to vascular calcification, hypertension, and preeclampsia [71–73], while SCFA supplementation or fiber-rich diets reduce blood pressure, cardiac fibrosis, and atherosclerosis [74–76].

SCFAs have been shown to exert their effects by binding to cognate G-protein-coupled receptors (GPRs), regulating the immune system and acting as epigenetic modifications of histone acetylate [77]. Recent studies also suggest that the cardioprotective role of SCFAs is mediated through these mechanisms. Specifically, SCFAs prevent the development of hypertension, cardiac hypertrophy and fibrosis via SCFA receptors, particularly GPR43 and GPR109A [75]. In *Apoe*^−/−^ and angiotensin II-induced models, propionate supplementation attenuates hypertension-associated cardiac hypertrophy through regulatory T (Treg) cell expansion [76]. SCFAs (especially butyrate) can function as histone deacetylase inhibitors (HDACi). In a streptozotocin (STZ)-induced diabetic model, butyrate, through its role of HDAC inhibition, improved myocardial function and attenuated cardiac hypertrophy in diabetic mice [78]. Notably, HDACi has been studied in preclinical models of CVD that shows decreased myocardial damage, improved cardiac function and myocardial repair [79–81]. Collectively, targeted interventions of SCFAs through direct supplementation or via precision modulation of SCFA-producing consortia may represent viable therapeutic approaches for CVD.

### Bile acids (BAs)

The gut microbiota plays a pivotal role in BA metabolism, converting liver-synthesized primary BAs into bioactive secondary BAs. Most primary and secondary BAs are actively reabsorbed in the terminal ileum and transported back to the liver via the portal vein, thus regulating enterohepatic circulation through nuclear receptors such as farnesoid X receptor (FXR) and G protein coupled receptor 1 (TGR5) [82]. Disorders in these BA metabolisms can cause cardiovascular and metabolic diseases such as dyslipidemia, diabetes, and fatty liver diseases [83]. BA receptors, including FXR and TGR5, have been recently proven to be expressed in cardiomyocytes, endothelial cells and vascular smooth muscle cells, indicating direct effects of BAs in CVD [84].

Clinical evidence shows that atrial fibrillation (AF) patients have significantly decreased serum ursodeoxycholic acid (UDCA) and increased hydrophobic BAs, with hydrophobic BAs identified as AF predictors [85]. Hydrophobic BAs, including lithocholic acid (LCA), deoxycholic acid (DCA), and chenodeoxycholic acid (CDCA), impair the cardiac mitochondrial function, accounting for cardiac alterations during cholestasis [86]. In contrast, UDCA exerts cardioprotective effects, preventing ischemia–reperfusion (IR) injury and cardiac infarction in isolated heart perfusion and in an anesthetized IR rat model [87, 88]. In high-fat diet (HFD)-fed mice, fructooligosaccharide leads to gut microbiota dysbiosis and abnormal BA enterohepatic circulation and thereby weakens the inhibition of FXR signaling pathway feedback [89]. Aspirin is commonly used to reduce the risk of adverse cardiovascular events, but its association with gastrointestinal damage is an increasing concern. *Parabacteroides goldsteinii* and its BA metabolite, 7-keto-lithocholic acid (7-keto-LCA), have been shown to reduce aspirin-mediated intestinal damage by acting as an FXR antagonist to suppress FXR signaling [90]. These studies highlight the role of intestinal BA metabolism in mediating drug–gut microbiota interactions. Detailed research is needed to elaborate on the perturbations of BA profiles associated with CVD pathogenesis.

### Phenylacetylglutamine (PAGln)

Beyond the aforementioned common metabolites, studies are beginning to identify novel metabolites associated with CVD. PAGln is a newly identified gut microbiota-derived metabolite that is involved in phenylalanine metabolism. Recently, plasma PAGln levels were found to be associated with CVD and incident MACCEs in large-scale clinical studies [54, 91]. In addition, PAGln has been shown to have prognostic value in patients with heart failure [92, 93]. Mechanistic studies revealed that PAGln directly enhances platelet activation and function through adrenergic receptors, including α2A, α2B, and β2 adrenergic receptors [54]. As PAGln is formed through the transformation of dietary phenylalanine into phenylacetic acid (PAA), characterizing the enzymes involved in this transformation is critical. Zhu et al. characterized two distinct gut microbial pathways for PAA formation: one that is executed by phenylpyruvate:ferredoxin oxidoreductase and the other by phenylpyruvate decarboxylase. The higher abundance of both pathways was confirmed in human atherosclerotic cardiovascular disease (ASCVD), suggesting that both genes contribute to an increased risk of ASCVD [94].

### Indoles and associated metabolites

Indoles, gut microbial metabolites of tryptophan, exhibit anti-inflammatory activity to improve intestinal health through affecting the host immune system via the aryl hydrocarbon receptor (AhR) or PXR signal pathway [95, 96]. Decreased serum indole-3-propionic acid (IPA) levels are linked to the risk of ASCVD as well as its severity. IPA supplementation in *Apoe*^−/−^ mice mitigates atherosclerotic plaque development by promoting macrophage reverse cholesterol transport through the miR-142-5p/ABCA1 pathway [97], and improves endothelial function and reverse antibiotic-induced vascular dilation via PXR activation [98]. Indole-3-carboxaldehyde (ICA) similarly alleviates atherosclerosis in HFD-fed mice by activating the AhR-Nrf2 pathway in endothelial cells [99]. However, there are also conflicting data that suggest indole, IPA and 5-OH-ICA are associated with incident (3-year) MACCE risk [91], indicating a bidirectional role of indoles in the cardiovascular system. This duality may stem from the chemical diversity of indole derivatives and their distinct receptor engagements. While physiological levels maintain vascular homeostasis, their pathological accumulation—especially in renal dysfunction—redirects signaling toward prothrombotic pathways. Instead of complete suppression, strategic modulation of the tryptophan metabolic flux may harness their therapeutic potential.

## Interactions between gut microbiota and TCM in CVD

TCM has been used extensively in China for thousands of years with a unique theoretical system and abundant practical experience. Emerging high-quality RCTs of TCM usage for CVD have also yielded promising cardioprotective outcomes. Despite these efforts, the underlying therapeutic mechanisms remain to be fully elucidated owing to the biological complexity of TCM.

**Fig. 2:**
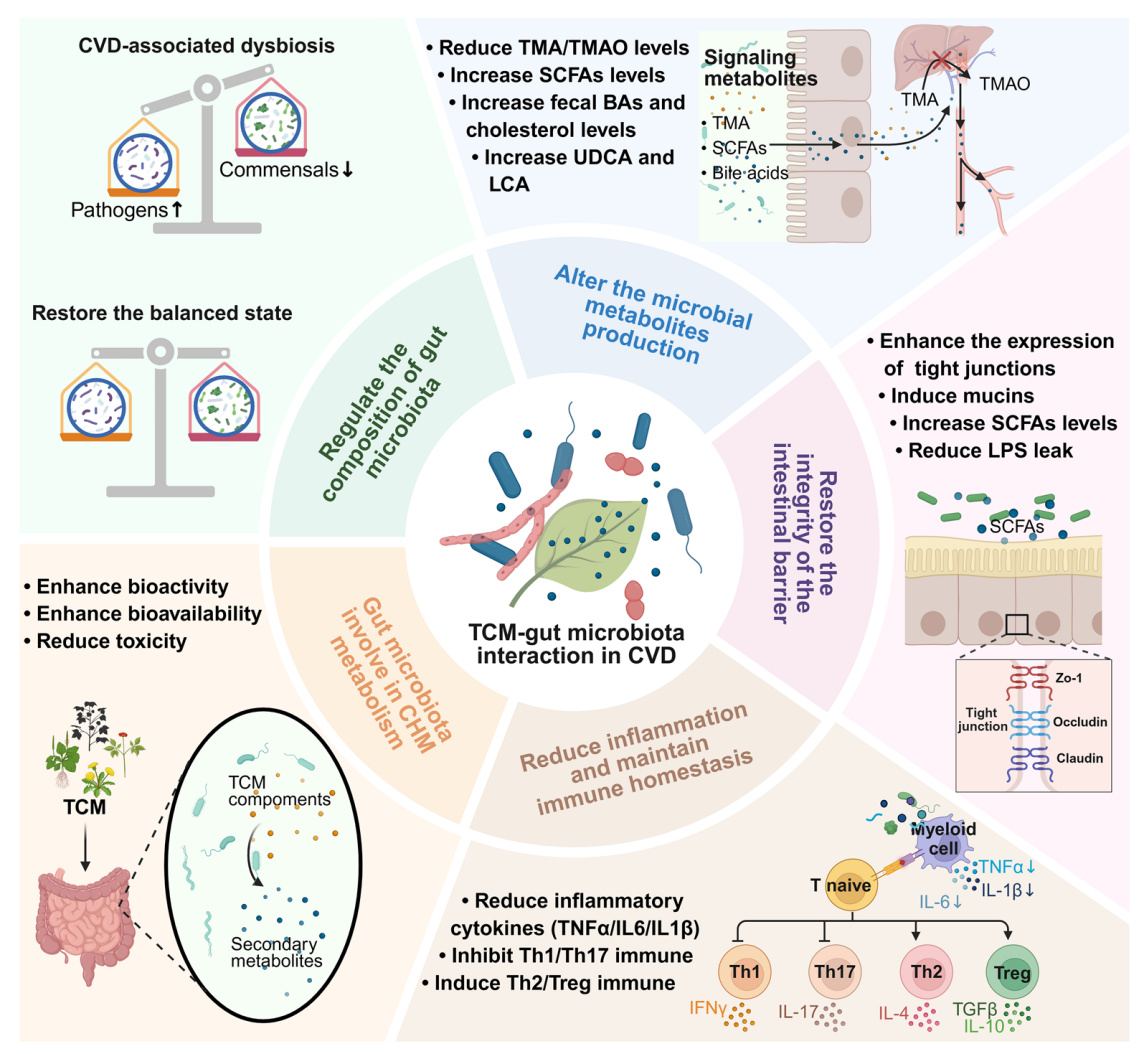
The interactions between TCM and gut microbiota in CVD. Gut microbiota is capable of metabolic effect on TCM. On the contrary, TCM can modulate gut microbiota to achieve its efficacy in treating CVD. Cardiovascular disease (CVD); Traditional Chinese Medicine (TCM); TMA (Trimethylamine); Trimethylamine-N-oxide (TMAO); Short-chain fatty acid (SCFA); Ursodeoxycholic acid (UDCA); Lithocholic acid (LCA); Lipopolysaccharide (LPS); Zonula occluden 1 (Zo-1); Regulatory T cell (Treg).

The gut microbiome has become a cutting edge area of research in the study of the associations among intrinsic (e.g., host genetics and diseases) factors, extrinsic factors (e.g., diets and medications), and host physiology [100]. Of note, the gut microbiota can influence drug structures and alter drug (including TCM) bioavailability, bioactivity, and toxicity (emergence of “pharmacomicrobiomics”); vice versa, the gut microbiota can be influenced by drugs [100]. Thus, an in-depth understanding of the microbiota–TCM interaction may pave the way for the modern scientific basis of TCM. Here, we provide a comprehensive overview of how gut microbes impact the TCM metabolism and how TCM affects the gut microbiota, as well as the underlying mechanisms in the treatment of CVD (Fig. 2). Furthermore, a gut microbiota–TCM interplay is proposed to be present in the treatment of drug-induced cardiotoxicity and is discussed.

### Metabolic effects of the gut microbiota on TCM

Pharmacomicrobiomics is an emerging area investigating the interactions between the microbiota and drug pharmacokinetics (absorption, distribution, metabolism and excretion) [101], which can facilitate probing the interplay between TCM and microbes. TCM comprises complex ingredients with a number of biological functions, including alkaloids, saponins, anthraquinones and flavonoids [22]. Gut microbial enzymes, including various carbohydrate-hydrolyzing enzymes, nitroreductase, azo reductase and glycosidases [102], can degrade TCM components into secondary chemical metabolites. Some metabolites have better bioavailabilities and bioactivities than their precursors. Glycosides (e.g., ginsenosides) primarily undergo deglycosylation via microbial glycosidases, enhancing their bioavailability and activity. Ginsenosides can metabolize to compound K (20-O-βd-glucopyranosyl-20(S)-protopanaxadiol) via the gut microbiota before being absorbed into the blood to achieve pharmacological functions [103]. Compound K has been shown to exhibit better bioavailability and increased antidiabetic and anti-inflammatory activities than its parent ginsenosides [104, 105]. Ginsenosides Rg1 [106] and Rb1 [107] have been proven to exert potential cardioprotective effects; therefore, the gut microbiota is clearly involved in the cardioprotective pharmacological action of ginseng. Alkaloids can be converted through microbial reduction into more absorbable forms. For example, berberine is a promising drug for the treatment of CVD owing to its multiple effects on the gut microbiome [108]. However, berberine exhibits poor water solubility and bioavailability. The gut microbiota can convert berberine into its absorbable form, dihydroberberine, which has a fivefold intestinal absorption rate compared to berberine. This compound oxidizes to berberine after absorption in intestinal tissues and subsequently enters the circulation [109]. Oxyberberine is a newly discovered gut microbiota-derived metabolite of berberine that has been shown to possess superior anti-colitis effects mediated by maintaining colonic integrity and inhibiting the inflammatory response [110]. As indicated above, an aberrant intestinal epithelial barrier and inflammation are linked to CVD, indicating that this is an underlying cardiovascular protective mechanism of berberine. The gut microbiota is also involved in the inactivation of TCM components. A special example is the inactivation of digoxin, a cardiac glycoside clinically used for heart failure and AF therapy, isolated from *Digitalis lanata*. Specifically, *Eubacterium lenta* was found to be responsible for digoxin inactivation [111], and antibiotic therapy could reverse the inactivation of digoxin by the gut flora [112]. In addition, the gut microbiota can reduce the toxicity of TCM. Low doses of aconitine have cardioprotective effects, but a narrow therapeutic window [113], and it can be metabolized by intestinal bacteria into benzoylaconine and lipoaconitine, which are substantially less toxic than their parent compounds [114]. We list the major TCM compounds transformed by the gut microbiota in Table 2.

**Table 2:** Transformation of TCM by gut microbiota

TCM compounds	Identified gut microbes	New metabolites	Effects on the precursors	Cardiovascular and cardiometabolic-related effects	References
Aconitine	*Bacteroides fragilis*, *Klebsiella pneumonia*,* Clostridium butyricum*	Benzoylaconine and lipoaconitine	Reduce toxicity	Protect myocardium (in low doses)	[115, 116]
Baicalin and baicalein	*Coryneum botulinum*,* Chaetomium spp.*	4’,5,6,7-tetrahydroxyflavone, 5,7dihydroxy-6-methoxxyflavone	Enhance bioavailability	Anti-inflammatory, antiatherogenic, antithrombotic	[117]
Berberine	*Bifidobacterium*, *Escherichia coli*,* Enterobacter cloacae and Enterococcus faecium*	Dihydroberberine (dhBBR)	Improve intestinal absorption	Reduce blood lipid	[109, 118]
Caffeic acid	*Eubacterium spp. strain* SDG-2, *Staphylococcus xylosus and Proteus mirabilis*	Dihydrocaffeic acid, mhydroxyphenylpropionic acid and M-Coumaric acid	Improve bioactivity and bioavailability	Prevent cardiovascular disease, improve vasodilation, lower blood pressure and anti-atherosclerosis	[119]
Catechin and epicatechin	*Clostridium cocoides*, *Eubacterium rectale group*	5-(3′,4′-dihydroxyphenyl)-γ valerolactone, 5-phenyl-γ valerolactone, phenylpropionic acid	Improve bioactivity and bioavailability, and act as prebiotics	Regulate blood lipids	[120]
Cholic acid	*Bacteroides intestinalis* AM-1	7-oxo-deoxycholic acid, 3,4-dichloroaniline (3,4-DCA)	Increase toxicity	Lower cholesterol	[121, 122]
Daidzin	*Bacteroides* J-37, *Eubacterium* A-44*, Fusobacterium* K-60	Daidzein, 2,4dihydroxyacetophenone, 4hydroxybenzoic acid, resorcinol, 4-hydroxyphenylacetic acid, 2,4-dihydroxybenzoic acid	Enhance bioavailability	Lower cholesterol	[123, 124]
Digoxin	*Eubacterium lenta*	Cardioinactive reduced metabolites including digoxin reduction product (DRP)	Inactivate	Increase myocardial contractility and slow heart rate	[125]
Dihydrodaidzein	*Eggerthella strain Julong* 732	(3S)-equol	Improve therapeutic effect	Improve endothelial function and Protect myocardium	[126]
Geniposide	*Bifidobacterium longum* HY8001*, Bacteroides fragilis*	Genipin	Increase toxicity	Inhibit platelet aggregation and thrombosis	[127, 128]
Ginsenosides Rb1, Rb2 and Rc	*Bifidobacterium* K-103, *Bifidobacterium* K-506, *Bifidobacterium sp.* Int57, *Bif. sp.* SJ32 and Int57, *Eubacterium* A-44, *Bacteroides* HJ-15, *Aspergillus niger*, *A. usamii*, *Esteya vermicola* CNU120806, *Paecilomyces bainier sp.* 229, *Fusarium sacchari*, *Fusarium moniliforme*, *Cladosporium cladosporioides*, *Acremonium strictum*, *Leuconostoc citreum* LH1, *Leuconostoc mesenteroides* DC102 *and Lactobacillus paralimentarius* LH4	Ginsenoside compound K (20-O-β-d-glucopyranosyl-20(S)-protopanaxadiol, CK)	Enhance bioavailability	Anti-inflammatory	[129–132]
Linoleic acid	*Butyrivibrio fibrisolvens*,* Clostridium proteoclasticum*	Rumenic acid (RA; cis-9, trans11-18:2), trans-9, trans-11–18:2, and trans-10, cis-12–18:2	Improve therapeutic effect	Regulate cholesterol levels and reduce atherosclerosis	[133, 134]
Naringin	*Bacteroides* JY-6, *Eubacterium* YK-4, *Feptostreptoccous* YK-10, *Fusobacterium* K-60, *Streptococcus* S-2, *Lactobacillus* L-2, *Bacteroides* JY-6	Naringenin, 4-hydroxybenzoic acid, phloroglucinol, 2,4,6-trihydroxybenzoic acid, 4hydroxyphenylacetic acid	Enhance bioavailability	Lower blood pressure and lipid levels	[123]
Pedunculagin	*Gordonibacter urolithinfaciens spp. nov*	Ellagic acid, urolithin A, B, C and D	Reduce side effects	Pedunculagin stimulates inflammation and angiogenesis and upregulate vascular endothelial growth factor and tumor necrosis factor-alpha	[135, 136]
Rhein	*Bacteroidetes*	NA	Improve therapeutic effect	Antidiabetic	[137]
Rutin	*Bacteroides* JY-6, *Fusobacterium* K-60*, Eubacterium* YK-4, *Streptococcus* S-2, *Lactobacillus* L-2, *Bacteroides* JY-6 *Bifidobacterium* B-9	Quercetin, 4-hydroxybenzoic acid; 3,4-dDihydroxybenzoic acid; 3,4-dihydroxyphenylacetic acid	Enhance bioavailability	Anti-myocardial damage, anti-platelet aggregation, improve heart remodeling	[123]
Saponins (geniposide, iridoid glycosides, flavone glycosides)	*Bacteroides*,* Firmicutes*	Secondary glycosides or aglycones	Enhance bioavailability	Anti-thrombosis	[138]
Secoisolariciresinol diglucoside	*Eptostreptococcus spp.* SDG-1, *Eubacterium spp.* SDG-2	(-)-secoisolariciresinol, 3demethyl-(-)-secoisolariciresinol, 2-(3-hydroxybenzyl)-3-(4hydroxy-3methoxybenzyl) butane-1, 4-diol, didemethylsecoisolariciresinol	Improve bioavailability	Regulate cholesterol levels and reduce atherosclerosis	[139]

NA: not applicable

### TCM influences the gut microbiota to achieve efficacy

Gut microbiota dysbiosis acts as an inevitable partner in the occurrence and development of CVD, conversely, CVD also leads to disruption of the gut microbiota. TCM can restore the relative balance in this microbiota disorder. To detail the regulatory role of TCM in the gut microbiota and its therapeutic mechanisms in CVD, we summarize its effects on CVD and related metabolic disorders from the perspective of the “gut–heart” axis (Table 3). The mechanisms of TCM for treating CVD by modulation of the gut microbiota involve the following: regulating the composition of the gut microbiota, modulating bacterial metabolites, enhancing intestinal barrier integrity, reducing inflammatory responses and maintaining immune homeostasis (Fig. 2).

**Table 3:** Effects of TCM on gut microbiota in treating CVD and related metabolic disorders

TCM	Gut microbial alterations	Associated mechanisms	Diseases/Animal models	References
Formulas				
Shenxiang Suhe pill (SXSH)	Increased: *Muribaculaceae, Allobaculum;* Decreased: *Lactobacillus*	Modulation of gut microbiota and serum metabolites, including downregulation of 2-n-tetrahydrothiophenecarboxylic acid and lysophosphatidylcholine	Acute myocardial infarction rat by ligating LAD coronary artery	[140]
Huoxue Wentong Formula (HX)	Decreased: *Bacteroides, Deferribacteres, Ruminococcus_sp._zagget7, Acidobacteria*	Related to valine, leucine, and isoleucine biosynthesis, fatty acid biosynthesis, and arachidonic acid metabolism pathways	Myocardial Ischemia rat by ligating LAD coronary artery	[141]
Baoyuan decoction (BYD)	*Firmicutes, Bacteroidetes*, and time-dependent alleviation of fecal metabolome disturbance	Related to amino acid metabolism, lipid metabolism, energy homeostasis, and oxidative stress pathways	Cardiac hypertrophy rats induced by chronic intraperitoneal infusion of ISO	[142, 143]
Fuzi decoction (FZD)	Increase in the *Firmicutes/Bacteroidetes* ratio and abundance of the *Lactobacillus*	Related to elevations in the SCFA content, and the regulation of valine, leucine, and isoleucine biosynthesis, galactose metabolism pathways	Chronic heart failure rats established by abdominal aortic coarctation	[144]
Guanxin Xiaoban capsules	Increased: *Akkermansia;* Decreased: *Faecalibaculum*	Inhibiting the AGE-RAGE signaling pathway	Atherosclerosis mice	[145]
Tanhuo Decoction (THD)	Decreased: LPS producing bacteria Increasing the complexity of gut microbiota, competition among LPS producing bacteria and opportunistic pathogenetic bacteria	Decreasing genes of TMA biosynthesis and increasing genes of TMA degradation	Randomized controlled trial in acute ischemic stroke patients	[146]
Tianma-Gouteng granules (TGG)	Increased: *Desulfovibio*, *Lachnoclostridium*, *Turicibacter;* Decreased: *Alluobaculum*,* Monoglobu*	Regulating bile acid metabolism; downregulating Farnesoid X Receptor and Fibroblast Growth Factor 15 levels in the liver and ileum; upregulating Cholesterol 7α-hydroxylase levels	Spontaneously hypertensive rats (SHR)	[147]
Suxiao Jiuxin pill (SJP)	Restored richness and diversity of gut microbiota	Remodeling the gut microbiota and host fatty acid metabolism	Acute myocardial infarction rats induced by isoproterenol	[148]
Ginseng Dingzhi Decoction (GN)	Increase in SCFAs-production bacteria and anti-inflammatory bacteria Decrease in pathogens	Regulating intestinal flora and maintaining mitochondrial homeostasis	Heart failure mice induced by transverse aortic constriction	[149]
Single herbs				
Ligustrum robustum (LR)	Increased: *Bifidobacterium;* Decreased: *Lachnospiraceae*, *Bacteroidales*, *Odoribacter*, *Oscillibacter*	Reducing TMA production, and increasing bile acid deconjugation and excretion	Atherosclerosis in *ApoE*^−/−^ mice	[150]
Gastrodia	Increased: *Bifidobacterium*, *Ligilactobacillus*, *Alloprevotella*; Decreased: *Bacteroides*,* Escheria-Shigella*	Restoration of gut microbiota, improvement of intestinal mucosal barrier, and reduction of inflammatory cytokines	Early atherosclerosis mice induced by high-fat diet	[151]
Monomers				
Fenugreek gum (FG), hawthorn pectin (HP), burdock inulin (BI)	Increase in the ratio of *Firmicutes* and *Bacteroidetes* and the abundance of beneficial bacteria	Reducing blood lipids by inhibiting cholesterol absorption, enhancing cholesterol excretion. Enhancing the production of SCFAs, especially butyric acid	Hyperlipidemia rats induced by high-fat diet	[152]
Tanshinone IIA	Improved gut microbiota α-diversity and β-diversity	Reducing intestinal damage, decreasing LPS release into serum, decreasing inflammation	Myocardial infarction mice by ligating LAD coronary artery	[153]
Paeonol (Pae)	Decreased abundance of gram-negative bacteria	Inhibiting gut microbial LPS accumulation, reducing monocyte/macrophage activation, inhibiting vascular smooth muscle cell proliferation	Atherosclerosis in *ApoE*^−/−^ mice	[154]
Resveratrol	Increase in the ratio of *Bacteroides* to *Firmicutes;* Increased: *Lactobacillus*, *Bifidobacterium*, *Bacteroides*, *Parabacteroides*; Decreased: *Enterococcus faecalis*, *Proteobacteria*, *Turicibacteraceae*, *Moryella*,* Lachnospiraceae*	Reducing TMA via inhibiting the metabolism of choline, decreasing genes expression related to fatty acid synthesis, lipogenesis and adipogenesis through the FiaF signaling pathway	Atherosclerosis in *ApoE*^−/−^ mice	[155, 156]
Berberine	Increased: *Akkermansia spp.*, *Allobaculum*, *Butyricoccus*, *Blautia*, *Bacteriodes*, *Phascolarctobacterium*, *Ruminococcus*, *Coprococcu*, *Lactobacillus*; Decreased: *C. sporogenes*, *A. hydrogenalis*, *Prevotella*,* Proteus*	Attenuate TMA/TMAO production and inhibit choline-to-TMA conversion	Atherosclerosis in *ApoE*^−/−^ mice	[157]
Astragaloside IV	Increased: *Bifidobacterium*, *Megamonas*, *Blautia*, *Holdemanella*, *Clostridium*	Reversing the autophagy and oxidative stress induced by the intestinal microbiota of acute ischemic stroke	FMT in germ-free mice with fecal supernatants of transient ischaemic attack and acute ischaemic stroke	[158]
Puerarin	Decreased: *Prevotella copri*	Inhibiting the production of TMA	Atherosclerosis in *ApoE*^−/−^ mice	[159]
Quercetin	Increased: *Actinobacteria*, *Cyanobacteria*, *Firmicute*s; Decreased: *Verrucomicrobia*	Reducing lipid levels and decreasing the levels of atherogenic lipid metabolites	Atherosclerosis in *Ldlr*^*−/−*^ mice	[160]

LAD: left anterior descending; ISO: isoproterenol; SCFA: short-chain fatty acid; AGE: advanced glycation end products; RAGE: receptor for advanced glycation end products; LPS: lipopolysaccharide; TMA: trimethylamine; ApoE: apolipoprotein E; Fiaf: fasting-induced adipose factor; TMAO: trimethylamine N-oxide; FMT: fecal microbiota transplantation; Ldlr: low-density lipoprotein receptor

#### Modulating the composition of the gut microbiota

TCM can directly impact microbiota promotion, inhibition, elimination, and colonization [114]. Some can inhibit the growth of specific bacteria, while others can stimulate the host immune system to release antimicrobial substances to avoid extraneous and indigenous pathogen colonization [161]. For example, cinnamon essential oil exhibits effective antibacterial activity against pathogenic *Escherichia coli*, *Staphylococcus aureus*, and *Pseudomonas deceptionensis* CM2 [162, 163]. Quercetin increases the relative abundance of beneficial bacteria, such as *Akkermansia*, *Lactobacillus aviaries* and *Faecalibaculum rodentium*, and decreases the abundance of *Parabacteroides*, *Alistipes*, *Musicspirillum*, and *Erysipelotrichaceae* [164–166]. By restoring the gut microbiome, quercetin ameliorates insulin resistance and metabolic disorders associated with CVD risk [164, 166]. Unsurprisingly, in a model of low-density lipoprotein receptor-null (*Ldlr*^−/−^) mice, quercetin was able to reduce lipid levels, as well as the extent of atherosclerotic lesions and plaque size [160]. Suxiao Jiuxin pills (SJPs) are a well-established patented TCM with definite cardiovascular-protective effects. In an isoproterenol-induced acute myocardial infarction rat model, SJPs restored the richness and diversity of the gut microbiota (including *Jeotgalicoccus*, *Lachnospiraceae*, and *Blautia*), suggesting that their protective effects against CVD were partly due to the modulation of the gut microbiota [148]. The above studies indicate that TCM can regulate the gut microbiota balance, restore the richness and diversity of the gut microbiota and thereby exert cardiovascular protective effects. In addition, TCM can not only directly inhibit the growth of pathogenic bacteria, but also increase the relative abundance of beneficial bacteria. Future research should clarify the direct interaction targets of TCM and the target microbiota to reveal the specific regulatory network between TCM and gut microbiota in CVD.

#### Influencing microbial metabolite production

Besides modulating the structure of the gut microbiota, TCM exerts significant regulatory effects on gut microbial metabolites, which constitutes another crucial mechanism of its cardiovascular protective actions. For instance, geraniin, a natural polyphenol, lowers the plasma TMAO level in *Apoe*^−/−^ atherosclerotic mice and attenuates atherosclerotic inflammation, with reduced interleukin (IL)-1β, IL-6, and tumor necrosis factor (TNF)-α levels [167]. Berberine attenuates TMA/TMAO production in C57BL/6 J and *Apoe*^−/−^ mice fed with a choline-supplemented chow diet and mitigates atherosclerotic lesion areas [157]. Berberine can also increase the amount of microorganisms in the gut microbiota that produce SCFAs, especially butyrate-producing bacteria that reduce plasma lipid and glucose levels [168]. Similarly, Fuzi decoction (FZD) has been shown to modulate SCFA production in a chronic heart failure model, with increased acetate, propionate, butyrate, and isopentanoic acid levels [144].

TCM also regulates gut bacteria and BA metabolism. Specifically, Alisa B 23-acetate (AB23A), a biologically active plant sterol, can increase fecal BA and cholesterol excretion to reduce the blood cholesterol level and can prevent atherosclerosis in ovariectomized mice by regulating FXR [169]. The pharmacological mechanism of quercetin for the treatment of metabolic syndrome-related diseases also involves the modulation of the gut microbiota–BA crosstalk [170]. Quercetin treatment substantially promotes the generation of UDCA and LCA, which bind to TGR5 on adipocytes and stimulate thermogenesis [170]. Naoxintong (NXT) capsules have shown therapeutic effects on CVD, alone or in combination with conventional interventions, in both clinical and basic studies without causing significant adverse events [171]. In a study of a high-fat diet (HFD) rat model, NXT exerted preventive effects on hyperlipidemia, and its effect was related to enhanced SCFA production and reduced bacterial bile salt hydrolase (BSH) activity [172]. Liguizhugan decoction can effectively improve insulin resistance in overweight/obese non-alcoholic steatohepatitis (NASH) patients, restore the microbial composition in NASH rats, along with metabolites such as TDCA, glutamic acid and isocaproic acid [173]. Together, the gut microbiota and its metabolites serve as indispensable therapeutic mechanisms for TCM in the treatment of CVD. In-depth studies are warranted to clarify how TCM precisely regulate metabolic enzymes or the gene expression of gut microbiota to affect TMAO, SCFA and BA synthesis, as well as to explore the downstream signaling pathways through which these metabolites interact with host cells (e.g., vascular endothelial cells, adipocytes) to exert cardiovascular protective effects.

#### Protecting the integrity of the intestinal barrier

The intestinal barrier, which consists of a multilayer barrier including mucus, epithelium, lamina propria and the gut vascular barrier, forms the largest interface between the external environment and the host [60]. As previously mentioned, an impaired intestinal barrier function is observed in CVD patients and is predictive of adverse cardiovascular outcomes. Accumulating evidence demonstrates that TCM exert cardioprotective effects by strengthening the gut barrier and reducing inflammation. Some TCM-derived compounds directly inhibit the infiltration of inflammatory monocytes and can reduce the secretion of pro-inflammatory cytokines such as TNF-α and IL-6, which alleviate mucosal inflammation and prevent intestinal barrier damage [174]. Vaccarin can alleviate inflammatory cytokines in intestinal tissue and serum; this action depends on improvements in gut microbiota dysbiosis and elevations in tight junction protein levels [175]. Quzhuo Tongbi decoction (QZTBD) can increase the abundance of butyrate-producing bacteria, resulting in enhanced intestinal barrier function in mice with metabolic diseases with upregulated expression of tight junction proteins [176]. Qushi Huayu decoction protects intestinal tight junctions and inhibits LPS gut leakage induced by a high-fat diet [176]. Kidney-tonifying blood-activating (KTBA) decoction has also been observed to delay cardiomyocyte hypertrophy and improve cardiac function by improving gut barrier function [177]. Previous studies have highlighted an increase in SCFA levels that contributes to gut barrier integrity and decreases serum LPS levels [178, 179]. Considering many TCM can promote the production of gut microbial SCFAs, their therapeutic mechanisms, although not fully examined, may involve restoring the “leaky gut”. This TCM-mediated intestinal mucosal barrier protection not only maintains the gut’s microecological balance but also mitigates low-grade systemic inflammation, highlighting the gut vascular barrier (GVB) as a novel TCM target in CVD.

### Gut microbiota-based traditional Chinese medicine syndrome differentiation

Syndrome differentiation, a unique feature of TCMs, is being increasingly validated through omics technologies. With multi-omics profiling of the gut microbiota, TCM’s theoretical frameworks (Qi–blood theory, yin–yang balance and meridian theory) are contextualized within modern biological systems. Metabolomic studies have also identified distinct metabolic profiles in different TCM syndromes [180]. Microbiota-based biomarkers are critical for TCM syndrome differentiation and predictions of treatment response. In patients with metabolic syndrome, specific microbial taxa and functional pathways are observed between phlegm-dampness syndrome and qi-yin deficiency syndrome groups [181]. A humid heat environment which may be associated with damp-heat syndrome, can cause gut microbiota dysbiosis (decreased abundance of *Lactobacillus murinus*) and increase the level of serum secondary BAs that ultimately trigger inflammation [182]. This suggests that TCM syndromes correspond to measurable biological differences, providing a bridge between traditional theory and modern diagnostics. Artificial intelligence (AI) and machine learning (ML) are transforming gut microbiota research by enabling the analysis of large, complex multi-omics datasets [183]. These advancements accelerate the translation of microbiota research into clinical practice from descriptive profiling to functional characterization, facilitating the identification of microbiota-based biomarkers that not only validate TCM syndrome differentiation in CVD but also enable personalized treatment strategies.

## The “TCM–gut–heart” axis in drug-induced cardiotoxicity

Drug-induced cardiotoxicity has gained increased attention with the development of new therapies, particularly for cancer—from chemical therapeutics to targeted molecular therapies and, most recently, immunotherapies [184]. Cardiotoxicity occurs after acute or chronic treatment and can damage cardiac tissues and disrupt heart electrophysiology [185]. Cardiotoxicity can be life-threatening; thus, many efforts have been made to seek effective cardioprotective strategies to avoid drug-induced cardiotoxicity.

Recent studies have implicated gut dysbiosis in cardiotoxicity as a new feature of the gut–heart axis. Doxorubicin (DOX), as one of the most effective chemotherapeutic agents, disrupts the intestinal epithelium, triggering the leakage of LPS into circulation and enhancing TLR4-mediated inflammation. Accordingly, strategies to deplete the gut microbiota or inhibit the TLR4 signaling pathway effectively alleviate DOX-induced toxicity [186]. Additionally, gut microbial-derived metabolites have been found directly impact drug metabolism and toxicity. For example, Li et al. showed that TMAO exacerbated DOX-induced murine cardiac fibrosis via the activation of the NLRP3 inflammasome in DOX-induced cardiac fibrosis. On the contrary, oral supplementation with butyrate-producing microbes, butyrate itself, butyrate derivatives, and butyrate prodrugs has been shown to effectively ameliorate cardiotoxicity [187–189]. Notably, the gut microbiota and its metabolites also influence the efficacy of cancer therapy, suggesting that the microbiota plays a central role in controlling responses to chemotherapy and immunotherapy [190–193]. Thus, modifying the gut microbiota may not only decrease cancer therapy-related cardiotoxicity but also improve therapy effectiveness. More efforts are needed to analyze the associations between different bacteria and the efficacy and toxicity of cancer therapy to bring the benefits of microbiota-targeting interventions to cardiotoxic cancer therapy.

TCM are rich in various chemical components, such as alkaloids, polysaccharides, polyphenols, isoflavones, and other active ingredients, with therapeutic value [194]. In recent studies, natural herbs have been used as adjuvants for cardioprotection against chemotherapy-induced cardiotoxicity [195]. For example, yellow wine polyphenolic compounds (YWPCs) ameliorate DOX-mediated inflammation and mitochondrial dysfunction by restoring DOX-induced gut dysbiosis [196]. Similarly, glabridin and emodin also reduce DOX-induced cardiotoxicity by remodeling the gut microbiota [197, 198]. The former also facilitates colonic macrophage M2 polarization, accompanied by downregulation of LPS and upregulation of butyrate [197]. Although cardio-oncology remains an emerging area, hopefully a deeper understanding of the complex “TCM–gut–heart” axis will provide insights into novel therapeutic approaches.

## Intergrating novel approaches for gut microbiota and TCM studies in CVD

Studies of the gut microbiota and TCM have been significantly enriched by emerging research paradigms and technologies. Advanced methodologies such as next-generation sequencing, multi-omics approaches and novel bioinformatics have enabled a deeper understanding of the complex interactions between the gut microbiota and host health, empowering researchers to identify specific target species for microbiota-based therapeutics [199]. In-depth research on the gut microbiota provides a new perspective for the modernization and clinical application of TCM in CVD. Firstly, it is essential to use multi-omics and bioinformatics technologies to decipher the mechanism by which TCM regulates the gut microbiota to improve CVD. Secondly, based on gut microbiota targets, TCM, with its rich resources and unique regulatory advantages, provides a broad space for the development of microbiota-targeted therapeutics. By establishing a high-throughput screening platform combining in vitro gut simulation systems, standardized microbial and metabolite libraries and small-molecule libraries, researchers can screen out TCM monomers, extracts or compound prescriptions that specifically regulate CVD’s key target bacteria from a large number of TCM resources. With the rapid development of biotechnology and synthetic biology, the next frontier is the engineering of more specific goal-directed microbes for human diseases, for example, targeting microbial enzymatic pathways. In particular, TMAO-lowering interventions are of considerable interest for their potential therapeutic benefit. Enzyme inhibitors (3,3-dimethyl-1-butanol) of TMA production, which target distinct microbial TMA lyases, efficiently inhibit atherosclerotic lesion development [200]. Another emerging specific targeting intervention is engineered bacteria. Indeed, administering the recombinant *Lactobacillus plantarum* NC8 strain, which produces angiotensin-converting enzyme inhibitory peptides, to spontaneously hypertensive rats led to a significant decrease in systolic blood pressure without side effects [201]. Thus, targeting causative gut microbial pathways may serve as a potential therapeutic strategy for TCM with regard to CVD treatment. Also, novel drug delivery systems such as hierarchical assemblies and drug-convertible carrier designs represent a novel paradigm for oral targeted therapies, providing a universal platform that can be adapted to load various therapeutics (e.g., small molecules, biologics, probiotics) [202]. This formulation exerts therapeutic effects via a three-pronged therapeutic strategy, which includes targeting pathological processes of intestinal inflammation, mucosal barrier damage and microbiota dysbiosis, laying a solid foundation for future TCM treatments in CVD.

Collectively, these advancements underscore the importance of integrating emerging technologies and research paradigms to uncover innovative mechanisms of the gut microbiota and TCM for treating CVD and to facilitate the development of next-generation precision therapies.

## Conclusions and perspectives

Increasing clinical and preclinical evidence is verifying the pivotal role of the microbiota in CVD, which extends the heart–gut axis hypothesis. In this review, we propose that the complex TCM–gut–heart axis is the scientific basis of TCM’s role in CVD treatment and management, offering insights to optimize current therapies. Despite the widespread use of TCM to treat CVD, the absence of clearly defined molecular targets and mechanistic pathways calls for further investigations into TCM–gut microbiota interactions. Various therapeutic methods that target the gut microbiota have shown promising results in clinical application, including probiotics, prebiotics, and fecal microbiota transplantation, which may also benefit CVD treatment. With the emergence of pharmacomicrobiology, research is being conducted into TCM to enhance their efficacy and bioavailability through modulating the gut microbiota. Meanwhile, TCM may exert regulatory roles in the microbiota to treat CVD gut dysbiosis. Building on these basic findings, future clinical trials should be designed that include longitudinal microbiome monitoring and pharmacomicrobiomic subtyping, allowing us to link microbial dynamics with treatment effects. A tiered strategy, from mechanistic deconstruction to hypothesis-driven clinical validation, will not only decode the scientific mechanisms of TCM but also facilitate therapeutics and preventions that precisely target the gut microbiota in CVD patients.

## Data Availability

No datasets were generated or analysed during the current study.

## Abbreviations

CVD: cardiovascular disease
TCM: Traditional Chinese Medicine
MACCE: major adverse cardiac and cerebrovascular event
SCFA: short-chain fatty acid
LPS: lipopolysaccharide
TLR4: Toll-like receptor 4
TMA: Trimethylamine
TMAO: Trimethylamine-N-oxide
GPR: G-protein-coupled receptor
Treg: regulatory T cell
HDACi: histone deacetylase inhibitor
STZ: streptozotocin
BA: bile acid
FXR: farnesoid X receptor
TGR5: G protein coupled receptor 1
AF: atrial fibrillation
UDCA: ursodeoxycholic acid
LCA: lithocholic acid
DCA: deoxycholic acid
CDCA: chenodeoxycholic acid
IR: ischemia–reperfusion
HFD: high-fat diet
PAGln: Phenylacetylglutamine
PAA: phenylacetic acid
ASVSD: atherosclerotic cardiovascular disease
AHR: aryl hydrocarbon receptor
IPA: indole-3-propionic acid
ICA: Indole-3-carboxaldehyde
SJP: Suxiao Jiuxin pill
IL: interleukin
TNF: tumor necrosis factor
FZD: Fuzi decoction
AB23A: Alisa B 23-acetate
NXT: Naoxintong
BSH: bile salt hydrolase
NASH: non-alcoholic steatohepatitis
QZTBD: Quzhuo Tongbi decoction
KTBA: Kidney-tonifying blood-activating
AI: artificial intelligence
ML: machine learning
DOX: doxorubicin
YWPC: yellow wine polyphenolic compounds
ZO-1: Zonula occluden 1
